# High-efficient serum-free differentiation of endothelial cells from human iPS cells

**DOI:** 10.1186/s13287-022-02924-x

**Published:** 2022-06-11

**Authors:** Sarkawt Hamad, Daniel Derichsweiler, John Antonydas Gaspar, Konrad Brockmeier, Jürgen Hescheler, Agapios Sachinidis, Kurt Paul Pfannkuche

**Affiliations:** 1 Medical Faculty, Center for Physiology and Pathophysiology, Institute for Neurophysiology, University of Cologne, Robert Koch Str. 39, 50931 Cologne, Germany; 2 Biology Department, Faculty of Science, Soran University, Kurdistan Region Soran, Iraq; 3 Department of Pediatric Cardiology, University Hospital of Cologne, Cologne, Germany; 4 Marga-and-Walter-Boll Laboratory for Cardiac Tissue Engineering, University of Cologne, Cologne, Germany; 5 Center for Molecular Medicine Cologne (CMMC), University of Cologne, Cologne, Germany

**Keywords:** Human induced pluripotent stem cells, iPS cells, hiPSCs, Differentiation, Endothelial cells, Regenerative medicine, 2D monolayer culture, 3D scalable bioreactor suspension culture, Wnt signaling, eNOS, LDL uptake, Vascular endothelium, Arterial endothelium, Angiogenesis

## Abstract

**Introduction:**

Endothelial cells (ECs) form the inner lining of all blood vessels of the body play important roles in vascular tone regulation, hormone secretion, anticoagulation, regulation of blood cell adhesion and immune cell extravasation. Limitless ECs sources are required to further in vitro investigations of ECs’ physiology and pathophysiology as well as for tissue engineering approaches. Ideally, the differentiation protocol avoids animal-derived components such as fetal serum and yields ECs at efficiencies that make further sorting obsolete for most applications.

**Method:**

Human induced pluripotent stem cells (hiPSCs) are cultured under serum-free conditions and induced into mesodermal progenitor cells via stimulation of Wnt signaling for 24 h. Mesodermal progenitor cells are further differentiated into ECs by utilizing a combination of human vascular endothelial growth factor A165 (VEGF), basic fibroblast growth factor (bFGF), 8-Bromoadenosine 3′,5′-cyclic monophosphate sodium salt monohydrate (8Bro) and melatonin (Mel) for 48 h.

**Result:**

This combination generates hiPSC derived ECs (hiPSC-ECs) at a fraction of 90.9 ± 1.5% and is easily transferable from the two-dimensional (2D) monolayer into three-dimensional (3D) scalable bioreactor suspension cultures. hiPSC-ECs are positive for CD31, VE-Cadherin, von Willebrand factor and CD34. Furthermore, the majority of hiPSC-ECs express the vascular endothelial marker CD184 (CXCR4).

**Conclusion:**

The differentiation method presented here generates hiPSC-ECs in only 6 days, without addition of animal sera and at high efficiency, hence providing a scalable source of hiPSC-ECs.

**Supplementary Information:**

The online version contains supplementary material available at 10.1186/s13287-022-02924-x.

## Introduction

Endothelial cells form the inner lining of all blood vessels of the mammalian organism. They regulate the permeability of capillaries, interact with immune cells, produce hormones and prevent coagulation [1, 2]. Therefore, ECs are of interest in regenerative medicine to support revascularization of ischemic tissues and to line artificial vascular structures in tissue engineering approaches [3]. Primary ECs that are isolated from human coronary arteries [4] and umbilical cord [5] represent a limited source, are associated with ethical issues, carry a potential risk for infection and have limited accessibility and proliferation [6].

In contrast, human induced pluripotent stem cells (hiPSCs) proliferate limitlessly and are capable of differentiating into various adult cell types [7], including ECs, providing a source to generate ECs in a scalable fashion and under standardized conditions [8, 9].

To date, several human pluripotent stem cell-derived endothelial cell (hiPSC-EC) differentiation protocols have been established: (1) The classical embryoid body approach generates ECs with an efficiency of 2–16% [10–12], and (2) the monolayer approach by culturing hiPSCs on matrices and controlled addition of growth factors increases the ratio of differentiation to 60–70%. Remaining obstacles include the high expense of differentiation media, use of animal-derived materials and the need for cell sorting [13–15]. Thus, the unmatched demand is a robust hiPSC-EC differentiation protocol, which is highly reproducible, cost-effective, omits animal-derived media compounds and yields highly pure ECs.

Several studies show that stimulation Wnt signaling for a short time with small molecules such as CHIR99021 results in mesodermal specification [16]. In vivo and in vitro experiments demonstrate that not only vascular endothelial cells growth factor (VEGF) enhances EC fate commitment, but also basic fibroblast growth factor (bFGF) further facilitates EC differentiation [17–19]. 8-Bromoadenosine 3′, 5′-cyclic monophosphate (8Bro) is an activator of cyclic AMP-dependent protein kinase, also known as protein kinase A (PKA), and was recently shown to support the differentiation of hiPSCs into ECs [20].

Melatonin (Mel) is a hormone secreted in the mammalian brain pineal gland. Melatonin acts via plasma membrane G-protein-coupled receptors and cytosolic receptors contributing to numerous cellular signaling pathways; in addition Mel works as a potent antioxidant to scavenge free radicals [21–24]. It is known that Mel interacts with VEGF signaling and shows an activity in modulating neovascularization [25, 26]. Hence, we hypothesized that manipulating Wnt signaling together with VEGF, bFGF, 8Bro and Mel improves differentiation of hiPSCs into human ECs above previously published protocols.

The aim of this study is to establish a robust hiPSC-ECs differentiation protocol that is cost-effective, highly reproducible, avoids use of animal-derived media compounds and yields highly pure human ECs.

## Materials and methods

### Human pluripotent stem cell culture

Human induced pluripotent stem cell line NP0040-8 (kindly provided by Dr. Tomo Saric, University of Cologne, Medical Faculty, Institute for Neurophysiology) was cultured on Matrigel Matrix (hESC-qualified, Corning, # 734–1440) pre-coated six-well plates at 10 μg / cm^2^ growth area in E8 medium consisting of DMEM/F12 (1:1) + Glutamax (Thermo Fisher Scientific, # 31,331–028) supplemented with 64 μg/ml L-ascorbic acid phosphate magnesium n-hydrate (Wako Chemicals Europe, # 013–12,061), 20 μg/ml insulin (Lilly Deutschland GmbH, “Humalog 100I.E.”), 5 μg/ml transferrin (Sigma-Aldrich, # T3705), 14 ng/mL sodium selenite (Sigma-Aldrich, # S5261), 100 ng/mL heparin sodium salt (Sigma-Aldrich, # H3149), 100 ng/mL basic fibroblast growth factor 2 (Peprotech, # 100-18B) and 2 ng/mL transforming growth factor β (Peprotech, # 100–21) in a humidified incubator at 37 °C with 5% CO_2_.

Every 3–4 days hiPSCs were sub-cultured when cell culture reached 70 – 80% confluency. The medium was removed, and the culture dish was washed by 1 mL DPBS (-/-), 1 mL ReLeSR™ (Stemcell Technologies, # 05,872) was added per well of six-well plates and incubated for 1 min at RT, the ReLeSR™ was removed, and the plate was incubated for another 2 min in a humidified incubator at 37 °C with 5% CO_2_. The plate was knocked against the bench horizontally to produce a uniform hiPSCs cluster suspension. 50–200 μL hiPSCs cluster suspension was transferred into fresh wells of Matrigel-coated six-well plates, 2 mL E8 medium per well was added supplemented with 5 μM of Rho Kinase (ROCK) inhibitor (Y27632, Adooq, # A11001-5), and the plate was incubated in a humidified incubator 37 °C with 5% CO_2_. Every 24–48 h a complete medium change was performed into E8 medium without ROCK.

### Generation of hiPSC-ECs in 2D monolayer culture

For differentiation of hiPSCs into ECs, a hiPSCs single cells suspension was prepared. Once cell culture reaches 70—80% culture confluency, the cell culture was washed by 1 mL DBPS (-/-), 1 mL ReLeSR™ per well of six-well plates was added, the plate was incubated in a humidified incubator at 37 °C with 5% CO_2_ for 7–10 min. After that, 2 mL E8 medium supplemented with 5 μM ROCK was added per well of a six-well plate. In order to form a cell suspension, the well content was pipetted up and down up to 10 times. Next, the cell suspension was filtered through a 40 μm cell strainer (Greiner Bio-One, # 542,040), centrifuged at 120 g, re-suspended into 1 mL E8 medium supplemented with 5 μM ROCK, and the cell number was counted by an automatic cell counter (NaNoEnTek, Korea). 0.021 × 10^6^ hiPSCs were added per cm^2^ growth area in 2 mL E8 medium supplemented with 5 μM ROCK per well of a six-well plate. This day was counted as day -2 and starting day of human endothelial cell differentiation, and hiPSCs were cultured in E8 medium from day − 2 for 48 h.

Next, on day 0, mesodermal induction was performed by a complete medium change into E6 medium supplemented with 6 μM CHIR99021 (LC Laboratories, # C-6556) for 24 h. The E6 medium composition was similar to E8 medium, but without supplementation of bFGF and TGFβ growth factors. On day 1, a complete medium change was performed into E6 without CHIR99021 supplementation.

Afterward, on day 2, endothelial induction was performed by a complete medium change into E6 supplemented with 300 ng / mL human vascular endothelial growth factor A165 (VEGF) (Peprotech, # 100–20), 200 ng / mL bFGF, 1 mM 8-Bromoadenosine 3′,5′-cyclic monophosphate sodium salt monohydrate (8Bro) (Sigma-Aldrich, # 1,002,183,637), and 50 μM Melatonin (Mel; Sigma-Aldrich, #M5250) for 48 h. From day 4 onward, the culture medium was changed every 48 h into E6 medium supplemented with 10 ng / mL VEGF and 10 ng / mL bFGF, and 10 μM hydrocortisone (Sigma-Aldrich, # H4001).

Later, on day 6, the culture dish was washed by 1 mL DPBS (-/-), and 1 mL 0.05% Trypsin–EDTA (Thermo Fisher Scientific, # 25,200–056) per well of a six-well plate, and the plate was then incubated in humidified incubator 37 °C with 5% CO_2_ for 10 min to dissociate EC cells. The ECs suspension was prepared by pipetting up to 10 times with a 1000 µL pipette. The ECs suspension was filtered through a 40 µm cell strainer, and ECs were counted by a fully automated counter with aid of trypan blue dye.

### Generation of hiPSC-ECs in 3D bioreactor culture

Bioreactor (DASGIP, Eppendorf) assembling and preparing has been described previously [27]. The 3D culture system was continuously agitated with 60 revolutions per minute (r.p.m.), 3 standard liter gas overlay per hour, 5% CO_2_ and 37 °C temperature from the beginning to the end of hiPSC-ECs differentiation. To differentiate hiPSC-ECs in a stirred bioreactor, 30 × 10^6^ NP0040 hiPSCs were inoculated in 100 mL E8 medium supplemented with 5 μM ROCK for 2 days. On day 0, culture medium was completely changed into 100 mL E6 medium supplemented with 6 μM CHIR99021 for 24 h. After 24 h, the culture medium was changed into E6 medium without CHIR99021 for another 24 h.

On day 2, the culture medium was refreshed with 100 mL E6 medium supplemented with 300 ng / mL human VEGF, 200 ng / mL bFGF, 1 mM 8Bro and 50 μM Mel for 48 h. From day 4 onward, the culture medium was changed into E6 medium supplemented with 10 ng / mL VEGF and 10 ng / mL bFGF for 48 h. On day 6, embryoid bodies of hiPSC-ECs (EB-ECs) were collected into 25 mL E6 medium for preparing EB-ECs suspension. 5 mL of EB-ECs suspension was used for dissociation of EB-ECs with 1 mL 0.05% Trypsin–EDTA per 2 mL EB-ECs suspension. Trypsin activity was blocked with 25% FBS in DMEM / F12; hiPSC-ECs suspension was filtered by a 40 µm strainer and counted.

### Characterization of hiPSC-ECs by flow cytometry

hiPSC-EC was dissociated into single cells, 0.25 × 10^6^ EC single cells were re-suspended into 0.5 mL of 0.5% BSA and 2 mM EDTA in DPBS (-/-) in a 1.5 mL Eppendorf tube and centrifuged at 300 g for 1 min at 4 °C, and supernatant was removed. The pellet was re-suspended in 50 μL of 0.5% BSA and 2 mM EDTA in DPBS (-/-) containing 1:50 of Anti-SSEA4-PE, human (Miltenyi Biotec, # 130–098-369) for characterizing hiPSC. For mesodermal cell characterization, single cells were fixed with 4% Paraformaldehyde (PFA; Polysciences, # 1884) in DPBS (-/-) and permeabilized with 0.5% t-Octylphenoxypolyethoxyethanol (Triton X-100; Sigma-Aldrich, # 9002–93-1) in DPBS (-/-). Cells were incubated with anti-brachyury (D2Z3J) rabbit mAb (Cell Signaling Technology, # 81,694). Secondary detection was done by 1:1000 dilution of Alexa 555 conjugated goat anti-rabbit IgG (Life technology, # A21430) for 20 min. 1:50 dilutions of α-human-CD31-APC (Miltenyi Biotec, # 130–110-670), α-human-VE-Cadherin-PE (Miltenyi Biotec, # 130–100-716), CD34-FITC, human (Miltenyi Biotec, # 130–113-178) and α-human-CD184-PE-Vio770 (Miltenyi Biotec, # 130–103-798) were used for characterization of ECs. After the staining process, 0.5 mL of 0.5% BSA and 2 mM EDTA in DPBS (-/-) were added and centrifuged and supernatant was removed. The pellet was re-suspended in 250 μL of 0.5% BSA and 2 mM EDTA in DPBS (-/-), and samples were stored at 4 °C in the dark until analysis. Prior analysis flow cytometer compensation was performed to prevent spillover of fluorochromes. Data were acquired by flow cytometry (LSR Fortessa Analyzer, BD Biosciences). FCSexpress 6 (De Novo Software, Glendale, CA) was used for data analyzation and graphical presentation.

### Characterization of hiPSC-ECs by immunofluorescent staining

0.2 × 10^6^ ECs were plated on 10 μg / cm^2^ Matrigel-coated round glass cover slips (Carl Roth, # D-76185) in 48-well plates (Greiner-Bio-One, # 677,180), maintained in E6 medium supplemented with 10 ng / mL VEGF, 10 ng / mL bFGF, 10 ng / mL EGF, 10 μM hydrocortisone (Sigma-Aldrich, # H4001), 50 μM Mel and incubated at 37 °C with 5% CO_2_ for 24 h.

The medium was removed, and ECs were fixed by 4% PFA in DPBS (-/-) for 10 min at RT. Fixed ECs were permeabilized by 0.5% Triton X-100 in DPBS (-/-) for 10 min at RT. The well with cover slip attached ECs was washed by 3% bovine serum albumin (BSA) (Sigma-Aldrich, # A2153) in DBPS (-/-), and ECs were blocked with 3% BSA in DPBS for 60 min at RT.

The blocking solution was removed, and 150 μL of 3% BSA was added, containing 1:150 dilution ratio of α-CD31-APC (Miltenyi Biotec, # 130–110-670), α-VE-Cadherin-AF488 (Santa Cruz, # sc9989) and α-vWF (Dako, # A008229-2) antibodies and 1:100 dilution ratio of Rabbit α-LDL receptor primary antibody (Abcam LDL uptake assay kit (cell-based), # ab133127), as well as 1:10 α-eNOS (BD bioscience, #610,297) against nitric oxide synthase overnight at 4 °C.

Following primary antibody binding, the well was washed twice with 3% BSA in DBPS (-/-) and 150 μL of 3% BSA was added containing 1:1000 Alexa Flour 555 goat α-rabbit (Life Technologies, # A21430), 1:100 DyLight 488-conjugated goat α-rabbit IgG secondary antibody (Abcam LDL uptake assay kit (cell-based), 1:100 Alexa Flour 555 goat α-mouse IgG1 (Life Technologies, # A21422) and Hoechst 33,342 for 60 min at RT in the dark. Three washing steps were performed with 3% BSA in DPBS. The cover slips with stained cells were taken out from plate and mounted upside down on glass slides with aid of 5 µL SlowFade™ Diamond Antifade Mountant (Life Technologies, # S36972). Finally, slides with attached cover slips were examined by confocal fluorescent microscopy (SP8, Leica) for obtaining images.

### Endothelial vascular tube formation

0.5 × 10^6^ HUVEC or hiPSC-ECs were re-suspended in 1 mL E6 medium supplemented with 10 ng / mL VEGF, 10 ng / mL bFGF, 10 ng / mL EGF, 10 μM hydrocortisone, 50 μM Mel and 15% FBS. 100 μL of cell suspensions was added into 96-well plates (Greiner Bio-One, # 655,180) pre-coated with 30 µL Matrigel per well, and 100 μL of E6 medium was added additionally. The plate was incubated for 24 h in humidified incubator at 37 °C with 5% CO_2_, and images were captured by light microscopy after 5 h and 24 h.

### Determination low-density lipoprotein uptake of hiPSC-ECs

0.25 × 10^6^ either HUVEC or hiPSC-ECs were seeded in 48-well plates on Matrigel-coated cover slips (Carl Roth, #219,827,535) in 0.5 mL E6 medium supplemented with 10 ng / mL VEGF, 10 ng / mL bFGF, 10 ng / mL EGF, 10 μM hydrocortisone and 50 μM Mel. After 24 h, 1 μL / 100 μL medium of LDL-DyLight 550 (Abcam LDL uptake assay kit, # ab133127) was added into the well that contained attached hiPSC-ECs or HUVEC separately, and the plate was incubated at 37 °C with 5% CO_2_ for 5 h.

Next, the culture medium was discarded, and the wells were washed with DPBS (-/-) and fixed by 4% PFA for 10 min. After fixation, cell permeabilization was performed by 0.5% Triton-X in DPBS (-/-) for 10 min as well as blocking that was performed by 3% BSA for 1 h. 1:100 rabbit α-LDL receptor primary antibody (Abcam LDL uptake assay kit) was then added and incubated overnight at 4–8 °C in the dark. Finally, 1:100 DyLight 488-conjugated goat α-rabbit IgG secondary antibody (Abcam LDL uptake assay kit) and 1.2 µg / mL Hoechst dye were added and incubated at RT for 30 min in the dark. Afterward, coverslips were embedded with 5 μL of SlowFade™ Diamond Antifade mounting medium on the glass slides and cover slips were evaluated by SP8 confocal microscopy.

### Determination of nitric oxide synthase expression

To determine endothelial cell nitric oxide synthase production, on day 6 of hiPSC-ECs differentiation, 0.25 × 10^6^ hiPSC-ECs parallel to HUVEC were seeded on 10 μg / cm^2^ Matrigel-coated coverslip in 48-well plates containing 0.5 mL E6 medium supplemented with 10 ng / mL VEGF, 10 ng / mL bFGF, 10 ng / mL EGF, 10 μM hydrocortisone, and 50 μM Mel. After 24 h, hiPSC-ECs were fixed by 4% PFA in DPBS for 10 min at RT, permeabilized by 0.5% Triton X-100 in DPBS (-/-) for 10 min at RT. Later, hiPSC-ECs were blocked with 3% BSA in DPBS (-/-) for 60 min at RT. After that, 1:10 α-eNOS (BD Bioscience, #610,297) in 3% BSA was added and incubated overnight at RT. Next, hiPSC-ECs were washed with DPBS (-/-) twice and stained by 1:100 Alexa Flour 555 goat α-mouse IgG1 and 1.2 µg / mL Hoechst dye in 3% BSA in DPBS (-/-) for 60 min at RT in the dark. At the end, hiPSC-ECs on cover slips were washed with DPBS three times, embedded and evaluated by confocal microscopy.

### Transcriptomic whole genome analysis

Total RNA from ECs (human induced pluripotent stem cell-derived endothelial cells, hiPSC-ECs; human coronary artery endothelial cells, HCAEC; human cardiac microvascular endothelial cells, HCMEC; human umbilical vein endothelial Cells, HUVEC; human saphenous vein endothelial cells, HSaVEC; human dermal microvascular endothelial cells, HDMEC; human pulmonary microvasculature endothelial cell, HPMEC) was isolated using the PureLink RNA Mini Kit (Life Technologies, # 12183018A). Samples of 10^6^ primary ECs each in RNAlater were obtained from PromoCell.

Briefly, 1 × 10^6^ ECs were added to RNase-free 1.5 tubes, washed with PBS (-/-) and centrifuged at 300 g for 2 min at 4 °C. The pellet was re-suspended in 1 ml TRIzol (Life Technologies, # 15,596,026). By repeated pipetting and mixing on a vortex mixer, the ECs were lysed for 30–60 s. The lysate was incubated at RT for 5 min. To separate the RNA from phenol, 0.2 ml chloroform (Sigma, # C-2432) was added per tube, mixed gently by hand and centrifuged at 12,000 g for 15 min at 4 °C. 350 μL of the colorless supernatants was transferred into new 1.5 ml RNase-free tubes, and 350 µL of absolute ethanol (Carl Roth, # 9065.3) was added per tube and vortexed well. 700 μL of the mixed sample was passed through a centrifuge cartridge with collection tubes (included in the kit) and centrifuged at 12,000 g for 1 min at RT. The centrifuge cartridge was washed with 500 µL wash buffer II (Life Technologies, #12183018A) and centrifuged at 12,000 g for 15 s at RT. 22 μL RNase-free water was used to elute RNA into 1.5 RNase-free tubes, and RNA aliquots were stored at − 80 °C.

For transcriptome analysis, 5.5 µg of fragmented biotin-labeled double-stranded cDNA from endothelial cells was hybridized on Clariom™ S arrays (Clariom™ S Arrays, Human Applied Biosystems by Thermo Fisher Scientific). After staining, the arrays were scanned with Affymetrix Gene-Chip Scanner-3000-7G, while quality control analysis was performed with Affymetrix GCOS software. Transcriptome analysis was performed at the Transcriptomics Core Facility of the Center for Molecular Medicine Cologne (CMMC).

The gene-level views of the human transcriptome with Clarion S Assays for the endothelial samples from different human cell types of our experiments were obtained. After the RMA normalization, only significantly expressed probesets were chosen using FDR F test. k-means cluster analysis was performed after transcript-wise normalization of signal values to a mean of 0 and an SD of 1 using Euclidean distance measurement, with the Cluster 3.0 tool from the Eisen laboratory [28]. The number of clusters was decided based on manual clustering for different values of k, varying k from 1 to 15 clusters. *K* = 12 was found optimal, while higher numbers of clusters other clusters did not result in a better separation of samples. The raw data are available at the NCBI GEO database (Home—GEO—NCBI (nih.gov)) under accession number GSE200399.

### Enzyme-Linked Immunosorbent Assay (ELISA) of eNOS

To validate eNOS expression of hiPSC-ECs, both NP0040 and NP0040-R (a transgenic subclone of NP0040 expressing mCherry) were differentiated into ECs with or without Mel supplementation from day 2 to day 4. Next, hiPSC-ECs were collected inside 1.5 mL reaction tubes on day 4 and day 6, respectively.

After that, cells were lysed with mammalian cell lysis kit (Sigma-Aldrich, # MCL1-1KT). eNOS was then detected in the lysate by ELISA (Human eNOS DeoSet ELISA; R&D systems, # DY950-05). An ELISA reader (Tecan, # 1,502,004,958) was used for quantification according to the manufacturer’s instructions.

### Sprouting EB-ECs assay

To observe capillary sprouting from EB-ECs, 0.3  ×  10^6^ cells either 789-O renal tumor cell line (ATCC® CRL-1932™) or NP0040-ECs per 1 mL E6 medium supplemented with 10 ng/mL VEGF, 10 ng/mL bFGF, 10 ng/mL EGF, 10 μM hydrocortisone, 50 μM Mel and 15% FBS were plated per well of a six-well plate separately. The plate was agitated at 40 r.p.m. and incubated for 24 h in a humidified incubator at 37 °C with 5% CO_2_. After 24 h, equal numbers of 789-O spheroids and hiPSC-ECs spheroids were pooled in 100 μL E6 medium supplemented with 10 ng/mL VEGF, 10 ng/mL bFGF, 10 ng/mL EGF, 10 μM hydrocortisone, 50 μM Mel and 15% FBS. The pooled spheroids were cultured on top of 50 μL Matrigel per well of a 96-well plate that was pre incubated at 37 °C with 5% CO_2_ for 1 h, and images were captured by light microscope after 1 min, 24 h, and 48 h.

### Three-dimensional (3D) culture of hiPSC-ECs and 789-O renal tumor spheroids (EBs)

NP0040 and 789-O cell lines were genetically engineered to express mCherry (NP0040-R) and green fluorescence proteins (789-O-GFP), respectively. NP0040-R was differentiated into ECs inside six-well plates by applying the 2D monolayer method. At day six of differentiation, NP0040-R-ECs were dissociated into single cells with 0.05% trypsin–EDTA. In parallel, 789-O-GFP cells were also dissociated with 0.05% trypsin–EDTA, filtered through a 40 µm cell strainer and counted.

NP0040-R-ECs and 789-O-GFP were re-suspended in 15% DMEM to achieve 20 µL cell suspension containing 1000 and 500 single cells, respectively. Drops of 20 µL cell suspension per drop were cultured over night as hanging drops on the lid of square tissue culture dishes to generate cell spheroids. Next, NP0040-R-ECs and 789-O-GFP spheroids were collected in a 15 mL falcon tube and spun down at 120 g for 1 min. Both types of spheroids were re-suspended in 20 µL ice-cold 15% DMEM. 60 µL of 4 °C cool Matrigel was then mixed with 20 µL EBs suspension using pre-chilled 100 µL pipette tips. Matrigel-spheroid suspension was poured into wells of a 96-well that were pre-coated 1 h before with 30 µL Matrigel per well, thereby forming a gel layer inside the wells. 45 min post-casting EBs-Matrigel suspension, 200 µL of 15% DMEM was added per well of the 96-well plate. The plate was incubated at 37 °C with 5% CO_2_. Every 24 h the plate was examined on a Carl Zeiss fluorescence microscope and/or SP8 Leica microscope.

### Statistical analysis

De Novo software version FCS Express 6 was used for flow cytometry analysis. GraphPad Prism software version 5 was used for graph drawing and statistical analysis. To find statistical difference between groups, one-way analysis of variance (ANOVA) was used, and Bonferroni's test also was used as a post hoc test. Data were represented as mean ± standard deviation (mean ± SD) when biological independent replications were three to six (*n* = 3–6) and significant difference value was less than 0.05 (*P* < 0.05).

### Data availability

The datasets generated during and/or analyzed during the current study are available from the corresponding author on reasonable request. The transcriptomics data are available as a supplementary file.

## Results

### Endothelial differentiation in 2D monolayer culture

**Fig. 1:**
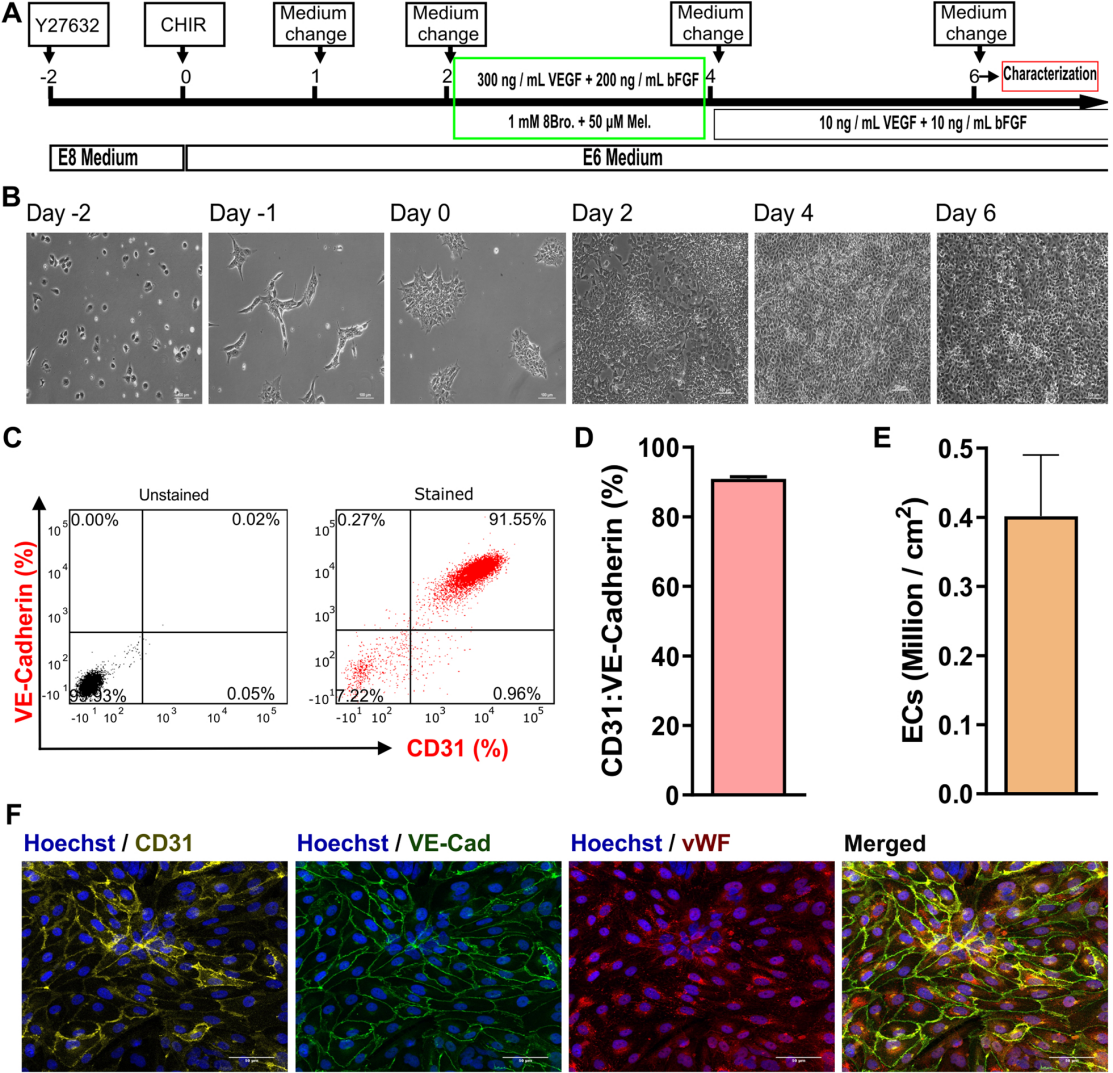
Endothelial cell differentiation in monolayer culture**. A** Schematic illustration of the differentiation protocol. **B** Bright-field images of different time points (day -2 to day 6) of hiPSC-ECs differentiation. Scale bar = 100 μm. **C** Representative flow cytometric scatter plots of negative control and hiPSC-ECs stained for CD31-APC:VE-Cadherin-PE endothelial markers. Ticker marks indicate bi-exponential presentation of the data. **D** Bar diagrams representing biological replicates (*n* = 6) of differentiated hiPSC-ECs on day 6, analyzed by flow cytometry for CD31-APC:VE-Cadherin-PE. **E** Numbers of hiPSC-ECs in million / cm.^2^ on day 6 (*n* = 6). **F** Immunostaining of hiPSC-ECs on day 6 of hiPSC-ECs differentiation for CD31-APC (yellow), VE-Cadherin-AlexaFluor488 (green), von Willebrand factor (red) and Hoechst dye (blue). Scale bar = 100 μm

Monolayer culture is suitable for testing many physiological hypotheses and is easily adaptable in the laboratory. Thus, we first established differentiation of hiPSCs toward hiPSC-ECs in a monolayer culture. To this end, hiPSCs were induced into ECs in two stages: firstly, induced into mesodermal cells by CHIR00921 from day 0 to day 1; secondly, into endothelial cells by VEGF, bFGF, 8Bro and Mel from day 2 to day 4 (48 h), and VEGF and bFGF from day 4 onward (Fig. 1A, and B). The concentration of CHIR99021 was optimized in two separate experiments (*n* = 3) for mesodermal and endothelial cell induction, and 6 μM CHIR99021 supplementation in E6 culture medium for 24 h was identified as an optimal concentration to induce hiPSC into mesodermal cells and then into hiPSC-ECs (Additional file 1: Figure S1 and Additional file 2: Figure S2). A CHIR 99021 concentration of 6 μM was selected for mesodermal induction due to the high percentage of hiPSC-ECs double positive for CD31:VE-Cadherin and the high yield of hiPSC-ECs per cm^2^ culture area.

On day 6 hiPSC-ECs were dissociated by 0.05% trypsin–EDTA and characterized by flow cytometry (FC) to detect the positive fraction of CD31:VE-Cadherin double positive ECs. FC analysis showed that 90.91 ± 1.52% of the cells (*n* = 7) were double positive for the endothelial markers CD31 and VE-Cadherin (Fig. 1C, D). FC analysis also confirmed that the presence of residual hiPSCs contaminations is in the range of background noise of the FC measurement (Figure S3). Numbers of hiPSC-ECs were calculated from the total number of live cells in millions / cm^2^, and the percentage of dual positive cells for CD31:VE-Caherin ECs markers resulting in a yield of 0.40 ± 0.09 million hiPSC-ECs generated per cm^2^ of culture surface (*n* = 7) (Fig. 1E). Immunocytochemical staining indicated that those monolayer differentiation cultures of hiPSC-ECs expressed the three ECs markers PECAM (CD31), VE-Cadherin (CD144) and von Willebrand factor (vWF) (Fig. 1F).

### Effects of different combinations of inducing factors on the differentiation of hiPSC-ECs

**Fig. 2:**
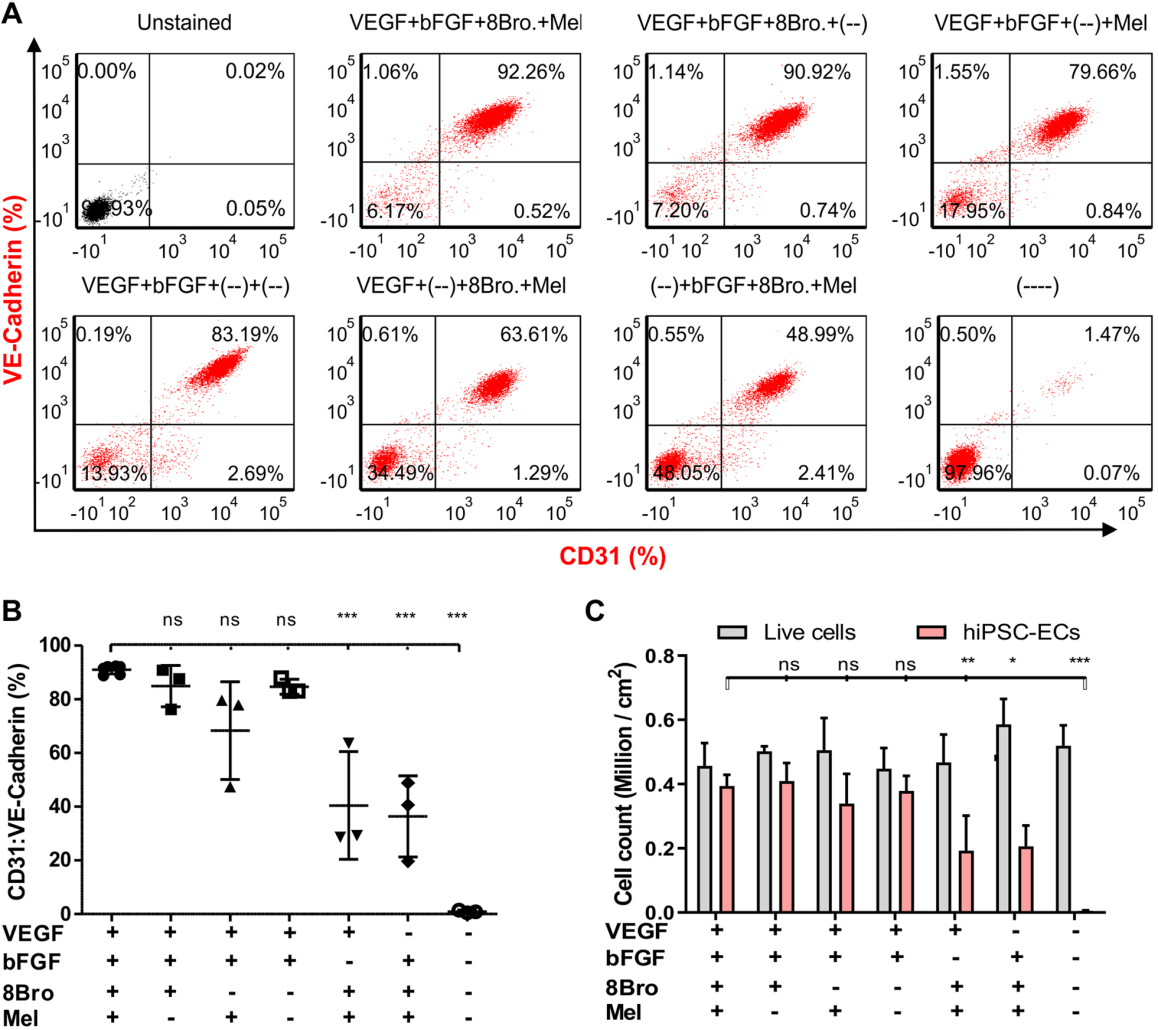
Effect of different combinations of growth factors, chemical compounds and melatonin on hiPSC-EC differentiation. **A** Representative scatter plot and **B** biological replicates (*n* = 6) of FC analysis on day 6. hiPSC-ECs differentiation was analyzed by staining for CD31-APC:VE-Cadherin-PE endothelial markers. **C** Total cell count (gray column) and number of hiPSC-ECs (red column) in million / cm^2^ on day 6, measured for six different settings of hiPSC-ECs induction. The number was depicted from the number of live cells (million / cm^2^) and the fraction of hiPSC-ECs double positive for CD31-APC:VE-Cadherin-PE. Data were expressed as mean ± S.D. *P* < 0.05 was considered as a significant difference versus control group

To identify the most efficient combination of inducing factors for robust differentiation, VEGF, bFGF, 8Bro and Mel, respectively, were tested in E6 medium. Combination of VEGF, bFGF, 8Bro and Mel from day 2 to day 4 followed by VEGF and bFGF from day 4 to day 6 of hiPSC-ECs differentiation resulted in 91.08 ± 1.59% hiPSC-ECs positive for CD31 and CD144 (*n* = 6). The negative control without addition of any of the aforementioned factors generated significantly less ECs (0.95 ± 0.49%, *n* = 3, *P* < 0.05) (Fig. 2A, B). The hiPSC-EC yield was determined as 0.39 ± 0.03 × 10^6^ ECs per cm^2^ (*n* = 6) in four-factor-induced hiPSC-EC differentiation (Fig. 2C).

Furthermore, to test for the presence of residual hiPSCs in the negative control group and the four-factor-induced hiPSC-ECs group, FC for SSEA4 was performed. FC analysis revealed that the percentage of SSEA4 positive cells was significantly (*P* < 0.05) reduced from day 0 (87.8% ± 1.59, *n* = 3) to day 6 of the differentiation process in differentiated cells without EC inducing factors (1.1 ± 0.31%, *n* = 4) and four-factor-induced hiPSC-ECs (1.9 ± 1.09%, *n* = 4), respectively **(**Additional file 3: Figure S3A, and B). The residual number of hiPSCs in million per cm^2^ significantly (*P* < 0.05) decreased from day 0 (0.06 ± 0.005) to day 6 in both differentiated cells without EC inducing factors (0.0051 ± 0.001, *n* = 4) and four-factor-induced hiPSC-ECs (0008 ± 0.005, *n* = 4), respectively **(**Additional file 3: Figure S3C, and D). The low number of hiPSCs in the absence of EC inducing factors indicates that E6 medium is not sufficient to support pluripotency.

### Characterization and functional analysis of hiPSC-ECs

**Fig. 3:**
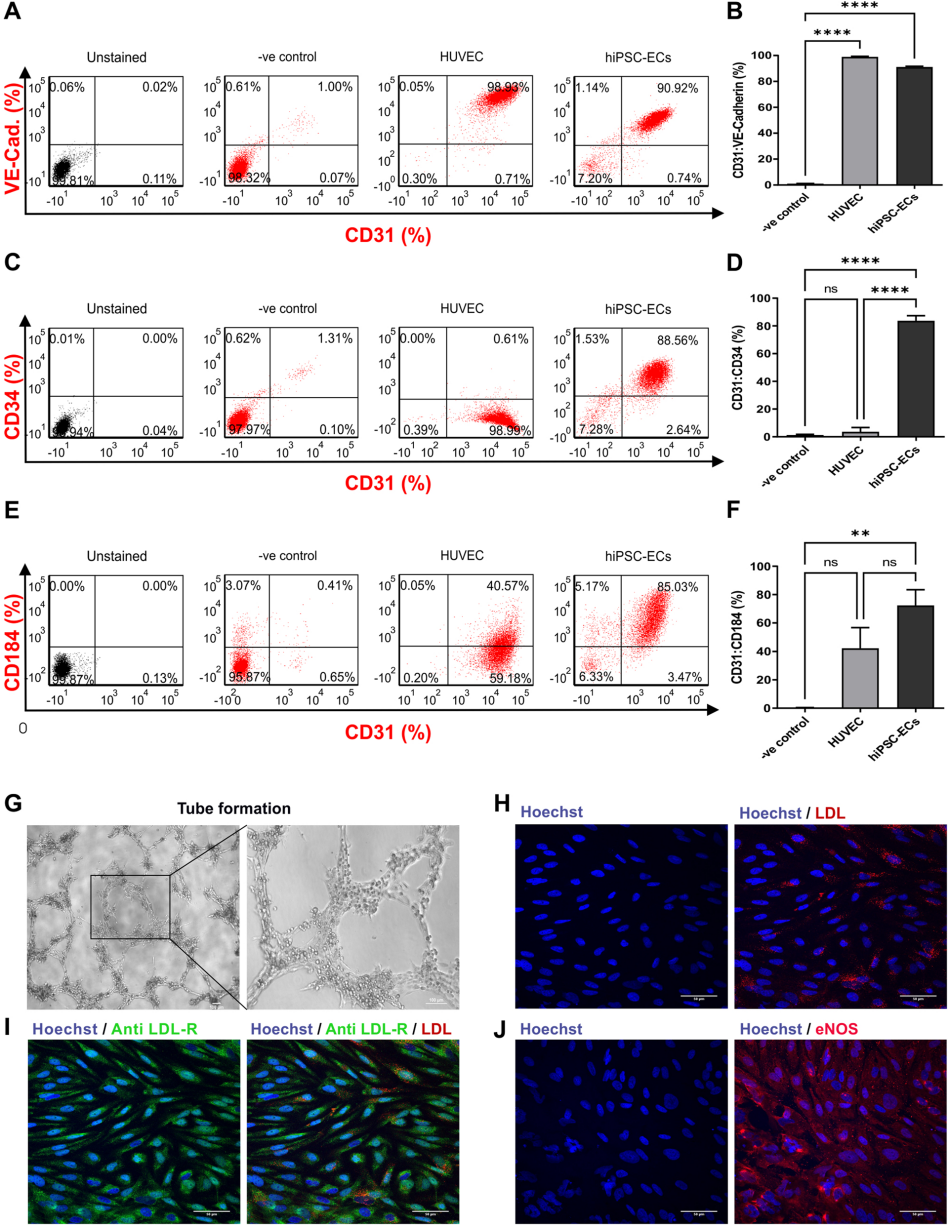
Characterization of hiPSC-ECs. **A**, **C** and **E** Representative plots, and **B**, **D**, and **F** biological replicates (*n* = 6) of FC analysis on day 6 of hiPSC-ECs differentiation for CD31-APC:VE-Cadherin-PE, CD31-APC:CD34-FITC and CD31-APC:CD184-PE double positive cells. Data were expressed mean ± S.D. *P* < 0.05 was considered as a significant difference versus control group. **G** hiPSC-ECs forming vascular tube-like structures on thick Matrigel layers. Images were obtained 5 h after seeding. Scale bar = 100 µm at 10X and 20X microscope lens power magnifications. **H** LDL uptake assessment of hiPSC-ECs. **I** Immunostaining with antibodies against LDL receptor (green). hiPSC-ECs were incubated with LDL-550 (red) for 5 h. **J** Staining with antibodies against nitric oxide synthase (anti-eNOS; red), and Hoechst against nuclei (blue). Scale bar = 100 μm

To further characterize the identity of hiPSC-ECs, flow cytometric analysis was performed for up to six biological replications of negative control and positive control (HUVEC) and hiPSC-ECs. FC revealed that HUVEC (98.9 ± 0.8%, *n* = 3) and hiPSC-ECs (91.08 ± 1.59%, *n* = 6) were significantly (*P* < 0.05) higher positive for CD31:VE-cadherin in comparison with the negative control (0.95 ± 0.49, *n* = 3) (Fig. 3 A and B). Additionally, hiPSC-EC contained 83.7% ± 7.42 (*n* = 4) of CD31:CD34 double positive cells and that ratio was significantly (*P* < 0.05) higher in comparison—VE control 3.57 ± 5.34% (*n* = 3) and HUVEC 1.19 ± 0.97% (*n* = 3), respectively (Fig. 3 C and D). hiPSC-ECs and HUVEC contained 72.28 ± 22.4% (*n* = 3) and 42.17 ± 25.13% (*n* = 3) of CD31:CD184 positive cells, and those ratios were significantly (*P* < 0.05) higher than the negative control 0.45 ± 0.07% (*n* = 3) (Fig. 3 E and F).

To determine cell functionality of HUVEC and hiPSC-ECs, different approaches were utilized including tube formation assay on Matrigel, LDL uptake assay, LDL receptor detection and nitric oxide (eNOS) synthase detection. In a tube formation assay, hiPSC-ECs and HUVEC were able to form vascular tube-like structures as early as after 5 h of incubation on thick layer Matrigel (Fig. 3G, and Additional file 4: Figure S4C).

To assess and detect LDL uptake, expression of LDL receptors and eNOS, single cells of both HUVEC and hiPSC-ECs were seeded on Matrigel-coated glass cover slips. Both, HUVEC and hiPSC-ECs, were found to be consistently positive for LDL uptake and anti-LDL receptor expression, respectively (Fig. 3H, I, and Additional file 4: Figure S4D, E), as well as positive for eNOS expression (Fig. 3J, and Additional file 4: Figure S4F). To further confirm eNOS expression, the ELISA technique was used to quantitatively determine eNOS protein in lysed cells at day 4 and day 6 of hiPSC-EC differentiation. ELISA data analysis showed that hiPSC-ECs expressed higher eNOS protein on day 4 (− Mel; 1605 ± 462, and + Mel; 1572 ± 301.7 *n* = 2), and day 6 (− Mel; 114.5 ± 38.89, and + Mel; 175.3 ± 33, *n* = 2) of hiPSC-ECs differentiation compared to the non-induced group (day 4; 162 ± 14.14, and day 6; 95.33 ± 51.85; *n* = 2), respectively (Additional file 5: Figure S5). The difference was significant only on day 4, while on day 6 there was only a non-significant trend toward higher eNOS expression in induced cells.

**Fig. 4:**
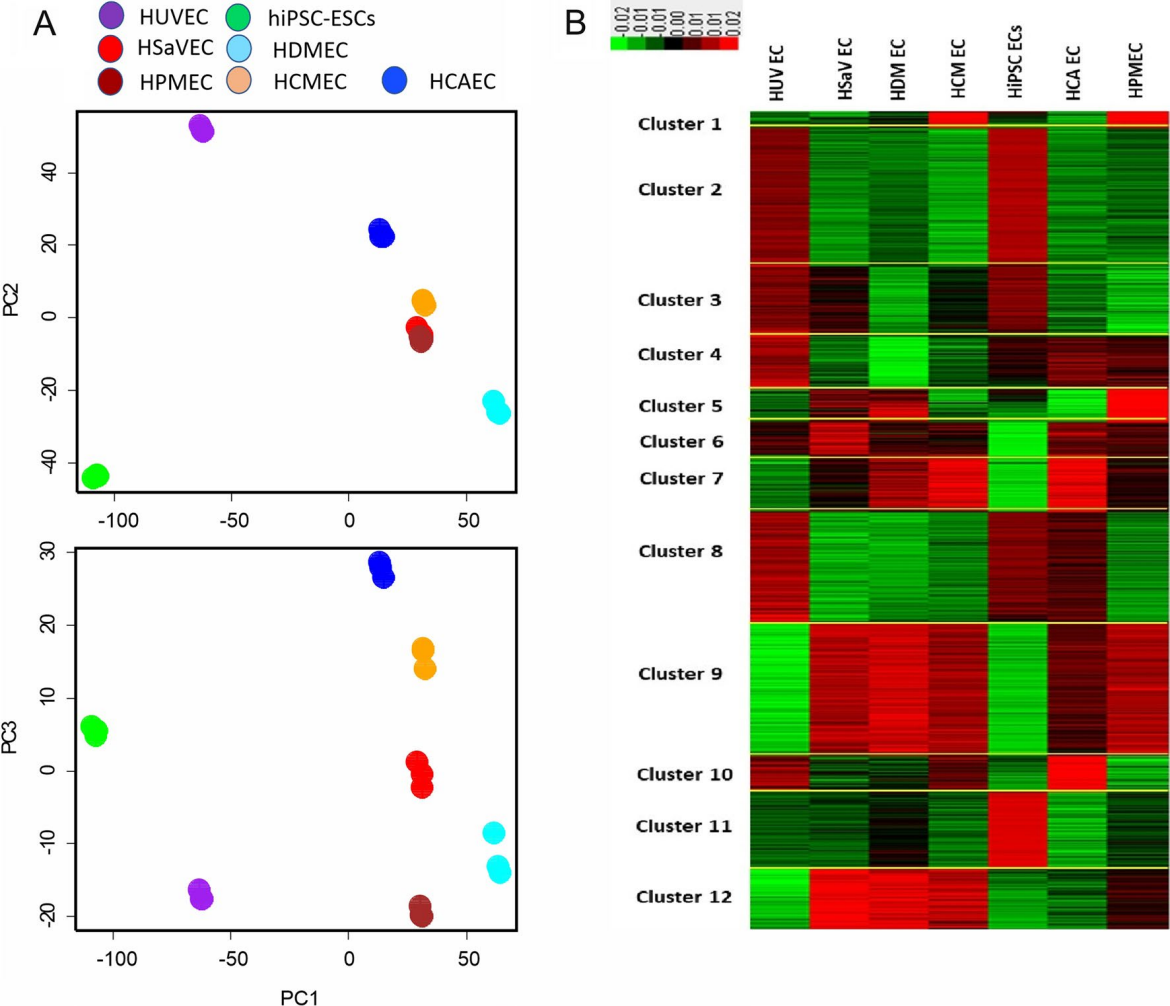
Transcriptomic analysis of endothelial cells. **A** Principle component analysis and **B** heatmap cluster analysis for transcriptomic data of human induced pluripotent stem cell-derived endothelial cells, hiPSC-ECs; human coronary artery endothelial cells, HCAEC; human cardiac microvasculature endothelial cells, HCMEC; human umbilical vein endothelial cells, HUVEC; human saphenous vein endothelial cells, HSaVEC; human dermal microvascular endothelial cells, HDMEC; human pulmonary microvasculature endothelial cell, HPMEC with one biological replications and three technical replications for each EC line

Principle component analysis of the whole genome transcriptomics of seven human endothelial cell types shows that hiPSC-ECs locate closer to HUVEC than to other tested primary ECs (Fig. 4A). K-Means-cluster analysis showed the twelve most prorated sub-clusters for the whole genome of seven human endothelial cells where each cluster shows specific differences for up- and downregulated genes (Fig. 4B, and Additional file 6: Table S1). Thus, Metascape bioinformatics [29] analysis has been used to detect the biological process within very low (cluster six) and high expression (cluster 11) gene express according to hiPSC-ECs. The Metascape analysis reveals that hiPSC-ECs have an early stage progenitor identity (Additional file 7: Figure S6A, and B). Specifically, genes grouped in cluster 11 were found upregulated in hiPSC-ECs compared to all other groups (Additional file 7: Figure S6B). The GOs found in cluster 11 can be mainly attributed to pathways of epithelial development. Hierarchical cluster analysis was performed to compare the expression profiles of all tested cell types. The cluster analysis is shown as a heatmap (Additional file 8: Figure S7) indicating that hiPSC-EBs cluster closer to HUVEC than other cell types. An analysis of transcript expression indicated a higher expression of eNOS in hiPSC-ECs as compared to 5 primary endothelial cell types (Additional file 5: Table S1, NOS3 gene). Only HUVEC showed a higher expression of eNOS.

### Sprouting

To show the response of hiPSC-ECs to tumor cells, in vitro ECs sprouting assays were performed. In a first experiment, spheroids from single cells of either hiPSC-ECs or 789-O tumor cells were generated. Spheroids of both EB-ECs and EB-789-O were pooled together on a Matrigel thick layer. Tumor cells were included to provide chemotactic factor inducing vessel sprouting. It was observed that Matrigel thick layer culture allowed formation of tube-like cell assemblies from EB-ECs, sprouting and branching toward EB-789-O within 24 h at 37 °C **(**Additional file 9: Figure S8.1). In a second experiment, hanging drop culture was used to form homogenous NP0040-R-ECs (hiPSC-ECs-R) and 789-O-GFP spheroids separately. Next, a 3D culture was established to monitor the growth of hiPSC-ECs in a 3D environment combining hiPSC-EC-R and 789-O-GFP EBs for nine days. Monitoring the 3D co-culture of hiPSC-ECs-R and 789-O-GFP EBs further confirmed that hiPSC-ECs-R generated branching tube-like structures toward 789-O-GFP EBs after 24 h. An increasing complexity of the structures was found during the observation period of 9 days (Additional file 10: Figure S8.2).

### Endothelial differentiation in 3D bioreactor culture

**Fig. 5:**
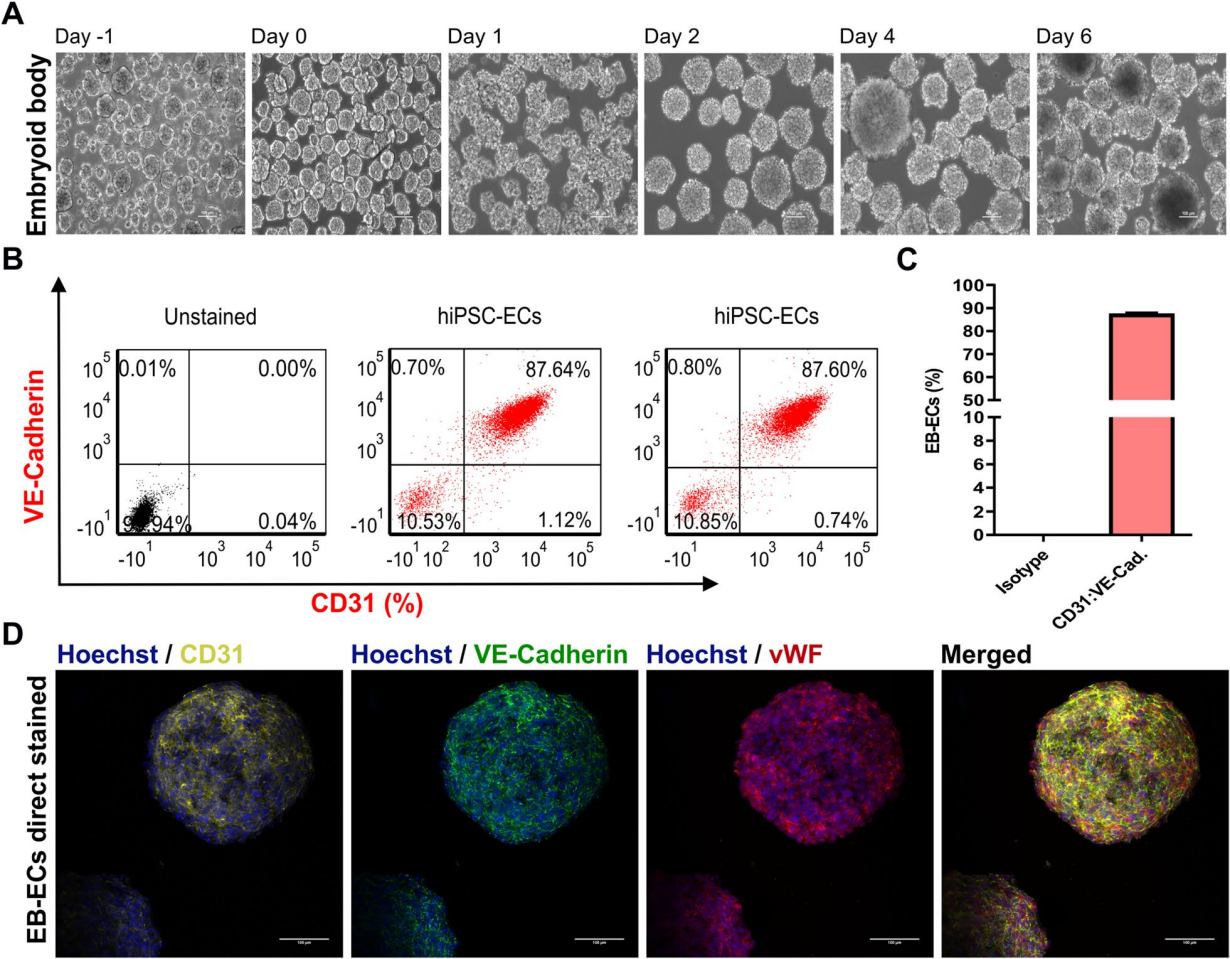
Differentiation of hiPSCs to endothelial cells in bioreactor suspension culture using the protocol optimized for 2D. **A** Bright-field images of cell aggregates formed in a suspension culture of NP0040 hiPSCs from day -4 to day 6 at 10X magnification. Scale bars: 100 μm. **B** Left panel scatter plot shows the fraction of CD31-APC:VE-Cardherin-PE positive cells at day 6 (red), analyzed by flow cytometry in 3 technical replications. Results of unstained controls are shown in gray. **C** Bar chart represents the fraction of positive cells as mean ± S.D. from 3 technical replications (isotype control shown in gray and staining shown in red). **D** Expression of CD31-APC (yellow), VE-Cadherin-AF488 (green) and von Willebrand factor (vWF; red), and Hoechst 33,342 against nuclei (blue) in hiPSC-ECs clusters at day 6 of differentiation. Images were obtained with a SP8 Leica confocal microscope. hiPSC-ECs clusters were stained as whole mounts. Scale bars: 100 μm

30 × 10^6^ hiPSCs were inoculated per 100 mL culture volume, and after embryoid body formation, the differentiation protocol established in 2D was applied. The protocol was sufficient to induce mesodermal specification and hiPSC-ECs-EBs (EB-ECs), respectively (Fig. 5A). As with monolayer differentiation, on day 6 EB-ECs were characterized by either FC or immunostaining. FC showed that EB-ECs contained 87% (*n* = 1) CD31:VE-Cadherin positive cells **(**Fig. 5B, C). Immunostaining confirmed that bioreactor-derived ECs were positive for CD31, VE-Cadherin and vWF (Fig. 5D).

For further characterization, EB-ECs were dissociated on day 6 into single cells, and different assays were performed. FC and immunocytochemistry analysis showed that EB-ECs generated in bioreactor were positive for several EC-specific protein markers, including CD31, CD34, CD184, VE-Cadherin and vWF (Additional file 11: Figure S9A, B). These EC-specific protein markers were also detected in both 2D culture derived hiPSC-ECs and HUVEC (Fig. 3, and Additional file 4: Figure S4). EB-ECs formed vascular tube-like structures on Matrigel after 5 h of cultivation (Additional file 11: Figure S9C). Furthermore, the bioreactor-derived EB-ECs were positive for LDL uptake, expressed LDL receptor (Additional file 11: Figure S9 D and E) and expressed eNOS (Additional file 11: Figure S9 F).

## Discussion

The protocol presented here promotes differentiation of hiPSCs into hiPSC-ECs, either in 2D monolayer or 3D scalable bioreactor suspension culture under serum-free conditions. Our new protocol confirmed that 24 h of Wnt signaling stimulation by the small molecule compound CHIR99021 alone is sufficient to differentiate hiPSCs into hiPSC-mesodermal cells (hiPSC-Meso). Furthermore, 48 h of inducing hiPSC-Meso with VEGF, bFGF, 8Bro and Mel turned out to be a robust method to differentiate hiPSC-Meso to hiPSC-ECs, providing > 90% of CD31:VE-Cadherin positive cells, and avoiding animal serum supplementation. Thereby, the protocol reduces the duration and the costs of hiPSC-EC production; it can be tuned for 2D and 3D culture systems with little effort.

Endothelial cells are derived from mesoderm, are required for formation of new blood vessels and are involved in vascular ton regulation in vivo [30–32]. In vitro studies proved that mesodermal cells can be differentiated from pluripotent stem cells and induced by bone morphogenic protein four (BMP 4), Activin A and bFGF either alone or in combination for 1–2 days together with Wnt signaling stimulation [33–36]. Utilizing recombinant growth factors such as BMP or Activin is associated with the risk of batch-to-batch variations and generates substantial costs. More recent in vitro studies indicated that stimulation of Wnt signaling by small molecules sufficiently induces mesodermal fate commitment [37, 38]. This strategy was applied in the present study, confirming that stimulation of Wnt signaling for 24 h via supplementing culture medium with the small molecule CHIR99021 is sufficient to generate up to 90% mesodermal cells. The mesodermal cell population induced by stimulation of Wnt signaling is dynamic and can be induced to develop into different cell types of the mesodermal origin in specific time windows with day 2 being the most suitable time for hiPSC-EC induction. The efficiency of the protocol presented here is dependent on hitting this time window of hiPSC-Meso development marking one of the major findings of our work.

In previous studies, the culture medium is supplemented with VEGF and bFGF either alone or in combination for further differentiating hiPSC-Meso into ECs [39–42]. Here, we show that both VEGF and bFGF are mandatory growth factors to induce hiPSC-Meso into ECs. Ikuno, T. and colleagues have found that activation of cAMP and PKA via 8Bro is supportive for hiPSC-ECs differentiation [20]. In our study, high concentrations of VEGF and bFGF synergistically promote the differentiation of hiPSC-ECs and further addition of 8Bro did not further enhance this effect. We have introduced Mel as a potential enhancer of EC commitment and found a reduction in inter-experimental variations when Mel was supplemented during hiPSC-EC differentiation induced by bFGF, VEGF and 8Bro. In contrast, in combination with bFGF and VEGF addition of Mel resulted in a trend toward higher variability.

It is known that Mel not only acts as an antioxidant, but also contributes to several cellular physiological pathways [43]. We found evidence that Mel increases eNOS expression in hiPSC-ECs at day 6 of differentiation. This finding is in line with a previous report showing an inducing effect of Mel on eNOS in HUVEC cells [44]. In conclusion, the four-factor differentiation with bFGF, VEGF, 8Bro and Mel resulted in a very low inter-experimental variation in our hand but did not show a significant advantage above induction with high dose of bFGF and VEGF.

Available protocols of ECs differentiation in 2D monolayer cultures result in an efficiency of ECs induction between 55 and 73%, including arterial and venous ECs. Fluorescence-activated cell sorting (FACS) or magnetically activated cell sorting (MACS) utilizing EC surface markers to eliminate unwanted side populations increases the 2D monolayer culture protocols efficiency up to 94% ECs purity [36, 45, 46]. In contrast, in our protocol synergistic stimulation of VEGF and bFGF pathways in combination with activation of PKA and Mel signaling improved the efficiency of EC differentiation to above 90% without cell sorting. Omitting one of the four factors VEGF, bFGF, 8Bro and Mel from the hiPSC-ECs differentiation process results in increased variations. Like others, our method of 2D differentiation hiPSC-ECs has limitations, including utilizing Matrigel for coating dishes that is prepared from mouse tumor cells [47].

To scale up hiPSC-ECs production, 3D bioreactor suspension culture systems have been used for forming EB-hiPSCs and further induce EB-hiPSCs into mesodermal and endothelial cells, respectively. Available approaches of 3D scalable EC differentiation protocols readily generate large numbers of ECs with up to 70% ECs purity; like 2D protocols FACS or MACS sorting technology increased the purity of ECs generated in 3D culture to above 96% [48, 49]. In comparison, our method of 2D monolayer hiPSC-ECs is easily applicable in 3D bioreactor suspension and yield hiPSC-ECs with up to 88% CD31 and VE-Cadherin positive cells without applying purification steps, while fully omitting serum and animal derived products during formation EBs and 3D suspension culture.

Recently, there have been doubts regarding the endothelial character of cells differentiated from human iPS cells. Lu and colleagues identified a misinterpretation of data in previous published work resulting in classifying hiPSC derivative epithelial cells as endothelial cells [50]. The cluster analysis of transcriptomic data (cluster 11) from our hiPSC-ECs revealed an upregulation of some epithelial cell development-related transcripts as compared to the bona fide ECs used for comparison. In the light of the work by Lu et al., we have critically evaluated our data to confirm that the protocol described here yields ECs and not epithelial cells. Lu et al. described a lack of eNOS and PECAM (CD31) expression in epithelial cells. In our study, we show a CD31 expression in more than 90% of the cells and found eNOS transcription comparable to primary ECs. Furthermore, eNOS expression was confirmed by immunocytochemistry and ELISA. The expression of epithelial marker protocadherin-10 was comparable to HUVEC cells, and keratin7 showed the lowest expression in hiPSC-ECs among all groups studied. Another marker indicative of epithelial expression according to Lu et al. is cadherin 1. We found significant expression of protocadherin 1 in all groups of endothelial cells. No difference (cutoff twofold changes) was found for cadherin 3. Tube-forming assay, expression of LDL receptor and LDL uptake provide additional evidence of endothelial commitment. Analysis of the hiPSC-ECs for marker proteins CD31, CD34, CD184 and VE-cadherin indicates a clear endothelial commitment as seen by high expression of CD31 and VE-cadherin but also reveals expression of CD34 indicating an immature state of the ECs. Comparison of hiPSC-ECs was performed against 6 different types of endothelial cells including 5 mature cell types and HUVECs on the basis of transcriptomics. The result shows closer relation of hiPSC-ECs to HUVEC cells as compared to mature EC cell types as shown by principal compound analysis and hierarchical cluster analysis. These findings further underline the premature state of the cells. We conclude that there is no evidence that the cells produced by the protocol described here belong to the family of ECs. However, the hiPSC-ECs have a potentially immature phenotype.

The optimized protocol can be seen as a basis for good manufacturing practice (GMP) conform production of hiPSC-ECs. In this work, we did not follow GMP guidelines for cell culture. In order to lift the process to GMP standards in the future, GMP qualified growth factors and small molecules should be used. Before transferring the process to GMP standards, attention has to be paid to the culture of the undifferentiated cells. Here, we used Matrigel as a culture substrate for expansion of cells in 2D what is not acceptable in a GMP process. Therefore, for pre-clinical and clinical applications of hiPSC-ECs is it advisable to replace Matrigel for recombinant GMP conform matrix proteins such as recombinant laminins. In the light of developing GMP conform protocols, the 2-factor induction with bFGF and VEGF could be favorable above the 4-factor approach including additional supplementation with 8Bro and Mel. Finally, as a limitation of the study it has to be mentioned that all experiments were conducted with the cell line NP0040, available from the European Bank for Induced Pluripotent Stem Cells, only. Further iPS cell lines, especially those derived from other types of somatic cells, may behave different and require further adaptation of the differentiation protocol.

## Conclusion

Synergistic induction of hiPSC derived mesodermal progenitors with VEGF, bFGF, 8Bro and Mel provides a robust method to differentiate hiPSC-ECs in only 6 days, without addition of animal sera. The differentiation efficiency of around 90% eliminates the need for further enrichment strategies in most experimental settings.

## Supplementary Information

**Additional file 1.** Fraction of t-box transcription factor brachyury positive progenitors in CHIR99021 induced cells.

**Additional file 2:** Effect of different concentrations of CHIR99021 on EC differentiation. A: Morphology of cell cultures treated with different concentrations of CHIR99021. B,C: Fraction of VE-Cadherin and CD31 positive ECs in cultures initially induced with different concentrations of CHIR99021.

**Additional file 3:** Quantification of remaining SSEA4 positive cells. SSEA4 positive cells were quantified by flow cytometry at the beginning of the differentiation protocol (day 0) and after 6 days in differentiation medium without addition of the four factors (-ve) or with addition of the four factors VEGF, bFGF, 8-Bro and Mel (+ve).

**Additional file 4.** Characterization of HUVEC cells. Figure S4 shows characterization of HUVEC cells to allow direct comparison with hiPSC-ECs (data for hiPSC-ECs is shown in Figure 3).

**Additional file 5.** ELISA quantification of eNOS expression in hiPSC-ECs at day4 and day6 of differentiation in presence or absence of melatonin.

**Additional file 6.** Transcriptomics of hiPSC-ECs in comparison to primary endothelial cells of different origin.

**Additional file 7.** Cluster 6 and Cluster 11 of metascape analysis. Metascape analysis revealed a cluster with genes downregulated (cluster 6) in hiPSC-ECs as compared to other EC types and a cluster of genes upregulated (cluster 11) as compared to other EC types. The figure summarizes the gene ontologies of the genes found in the respective clusters.

**Additional file 8**. Heatmap of transcriptomic profiling of different types of endothelial cells.

**Additional file 9.** Morphology of hiPSC-EC clusters in 3D culture.

**Additional file 10.** Morphology of red fluorescent hiPSC-EC clusters cultivated in a 3D cell culture model together with green fluorescent renal carcinoma cells.

**Additional file 11:**Analysis of hiPSC-ECs generated in a 3D bioreactor.

## Data Availability

Transcriptomic data are provided as supplementary information. The raw data are available at NCBI GEO database (Home—GEO—NCBI (nih.gov)) under accession number GSE200399.

## Abbreviations

8Bro: 8-Bromoadenosine 3′,5′-cyclic monophosphate sodium salt monohydrate
bFGF: Basic fibroblast growth factor
BSA: Bovine serum albumin
DPBS: Dulbecco´s phosphate buffered saline
DMEM: Dulbecco´s modified eagle medium
ECs: Endothelial cells
eNOS: Endothelial nitric oxide synthase
GO: Gene ontology
HCAEC: Human coronary artery endothelial cells
HCMEC: Human cardiac microvascular endothelial cells
HDMEC: Human dermal microvascular endothelial cells
hiPSC-ECs: Human induced pluripotent stem cell-derived ECs
hiPSC-Meso: Human induced pluripotent stem cell-derived mesodermal progenitor cells
HPMEC: Human pulmonary microvasculature endothelial cell
HSaVEC: Human saphenous vein endothelial cells
HUVEC: Human umbilical vein endothelial cells
iPSCs: Induced pluripotent stem cells
LDL: Low-density lipoprotein
Mel: Melatonin
PFA: Paraformaldehyde
PKA: Protein kinase A
ROCK: Rho kinase inhibitor
RT: Room temperature
VEGF: Vascular endothelial growth factor A165
vWF: Von Willebrand factor
