# Supplementary figures and images for: High-efficient serum-free differentiation of endothelial cells from human iPS cells
Source: Stem Cell Res Ther. 2022 Jun 11;13:251. doi: 10.1186/s13287-022-02924-x (PMC9188069; doi:10.1186/s13287-022-02924-x)

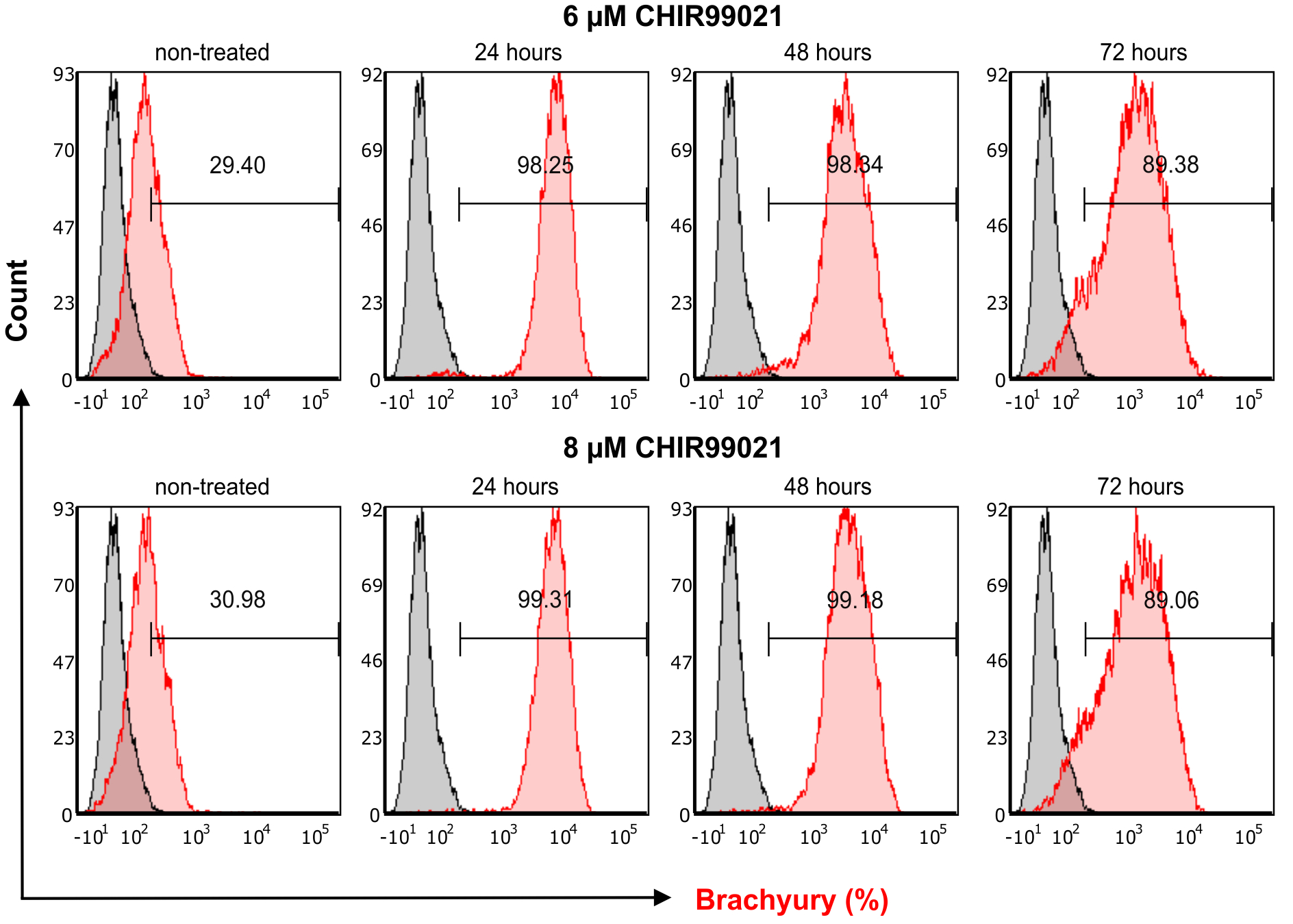

Supplement: Supplementary file 1 — Additional file 1. Fraction of t-box transcription factor brachyury positive progenitors in CHIR99021 induced cells. [file 13287_2022_2924_MOESM1_ESM.jpg]

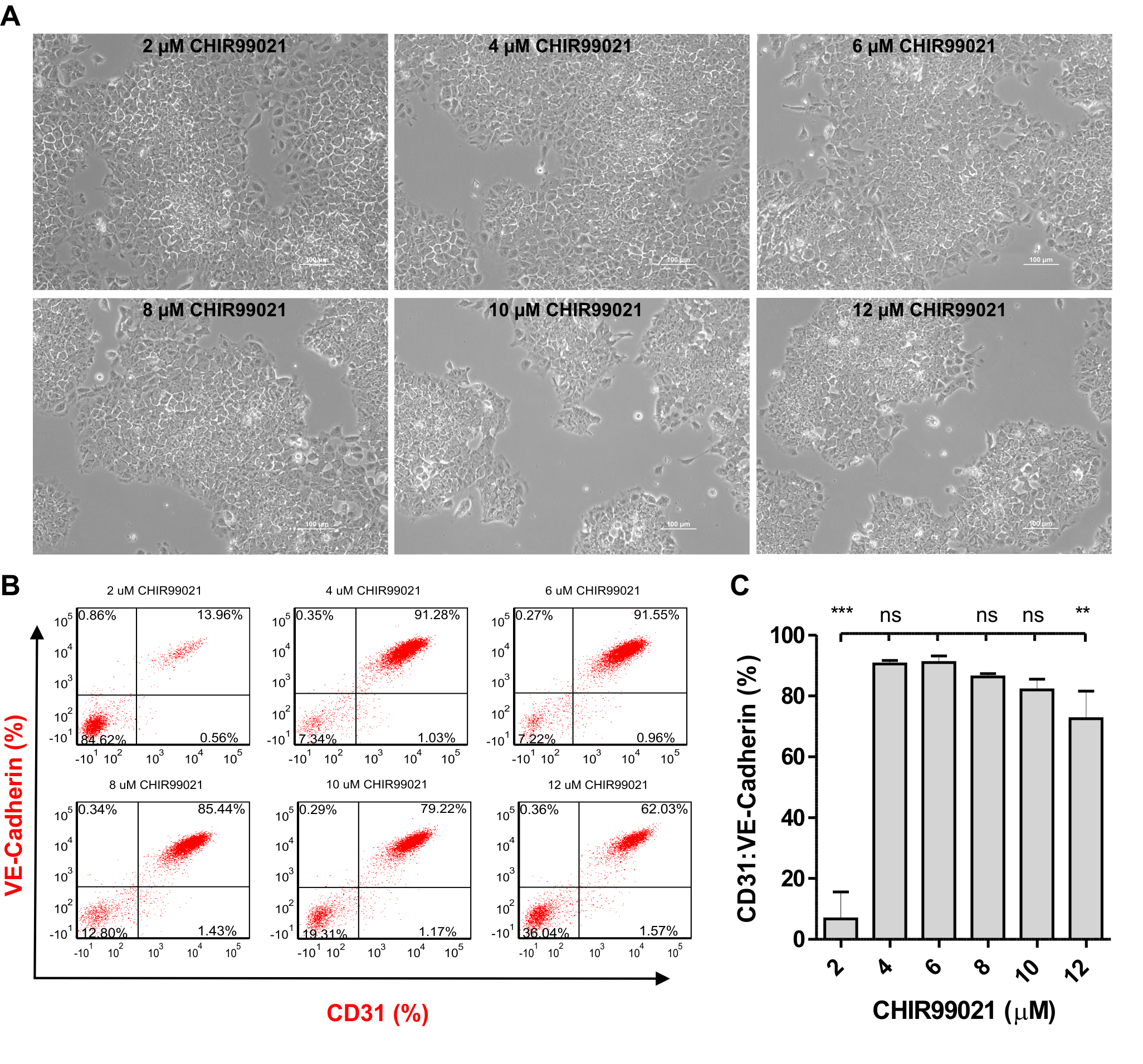

Supplement: Supplementary file 2 — Additional file 2: Effect of different concentrations of CHIR99021 on EC differentiation. A: Morphology of cell cultures treated with different concentrations of CHIR99021. B,C: Fraction of VE-Cadherin and CD31 positive ECs in cultures initially induced with different concentrations of CHIR99021. [file 13287_2022_2924_MOESM2_ESM.jpg]

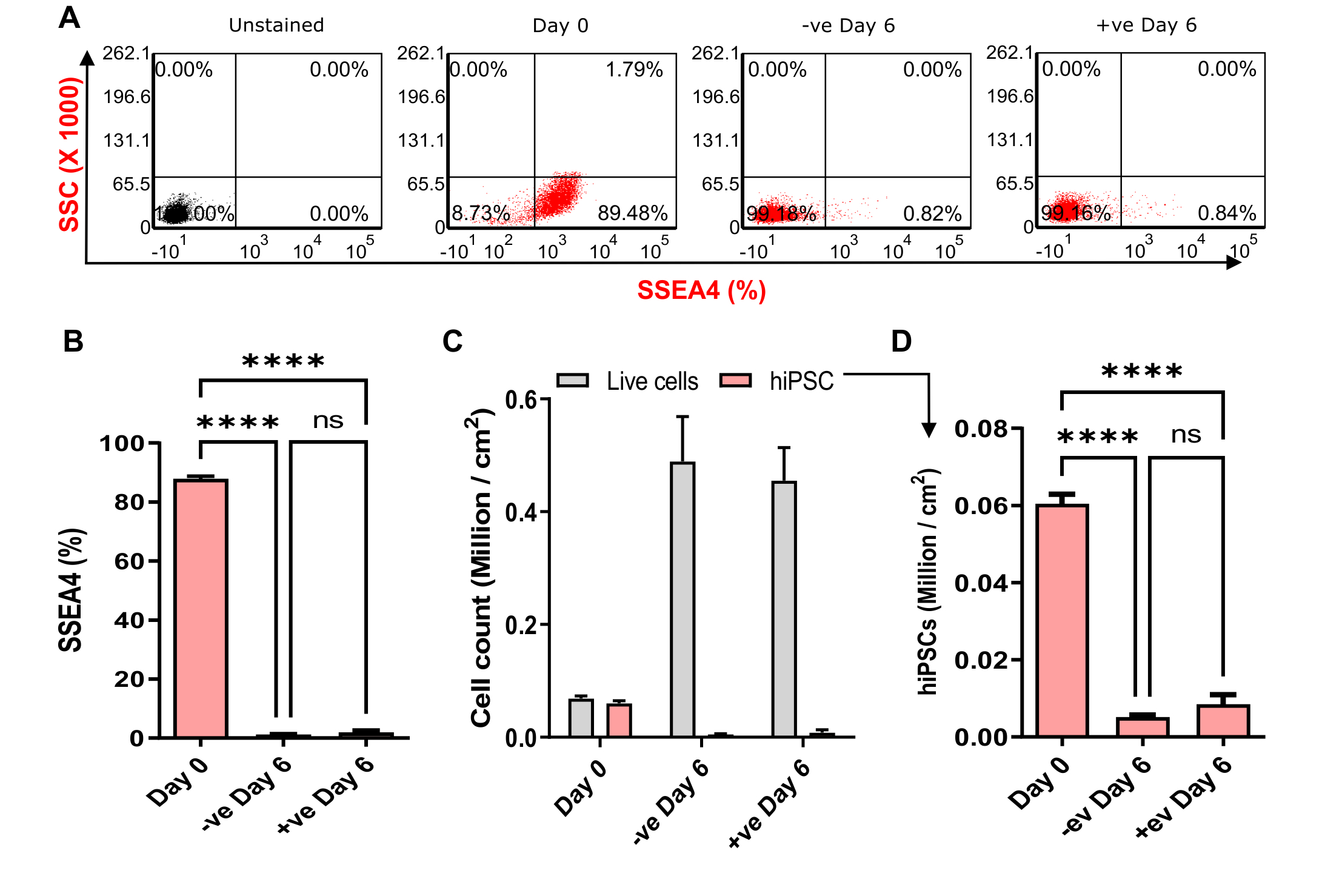

Supplement: Supplementary file 3 — Additional file 3: Quantification of remaining SSEA4 positive cells. SSEA4 positive cells were quantified by flow cytometry at the beginning of the differentiation protocol (day 0) and after 6 days in differentiation medium without addition of the four factors (-ve) or with addition of the four factors VEGF, bFGF, 8-Bro and Mel (+ve). [file 13287_2022_2924_MOESM3_ESM.jpg]

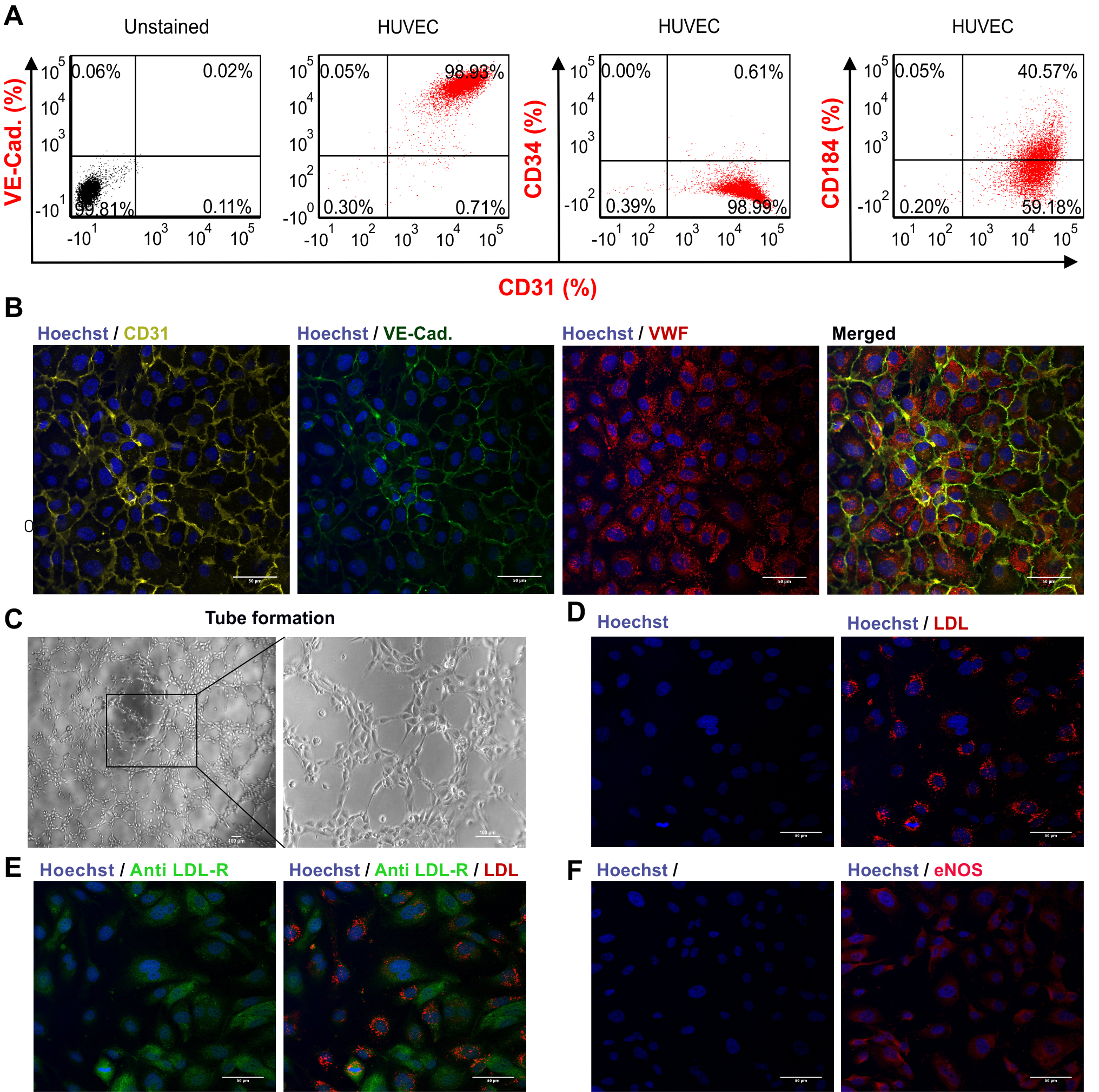

Supplement: Supplementary file 4 — Additional file 4. Characterization of HUVEC cells. Figure S4 shows characterization of HUVEC cells to allow direct comparison with hiPSC-ECs (data for hiPSC-ECs is shown in Figure 3). [file 13287_2022_2924_MOESM4_ESM.jpg]

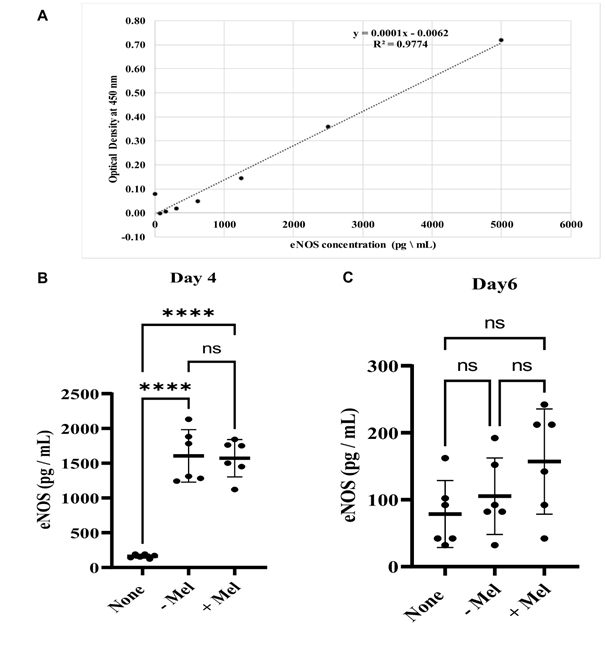

Supplement: Supplementary file 5 — Additional file 5. ELISA quantification of eNOS expression in hiPSC-ECs at day4 and day6 of differentiation in presence or absence of melatonin. [file 13287_2022_2924_MOESM5_ESM.jpg]

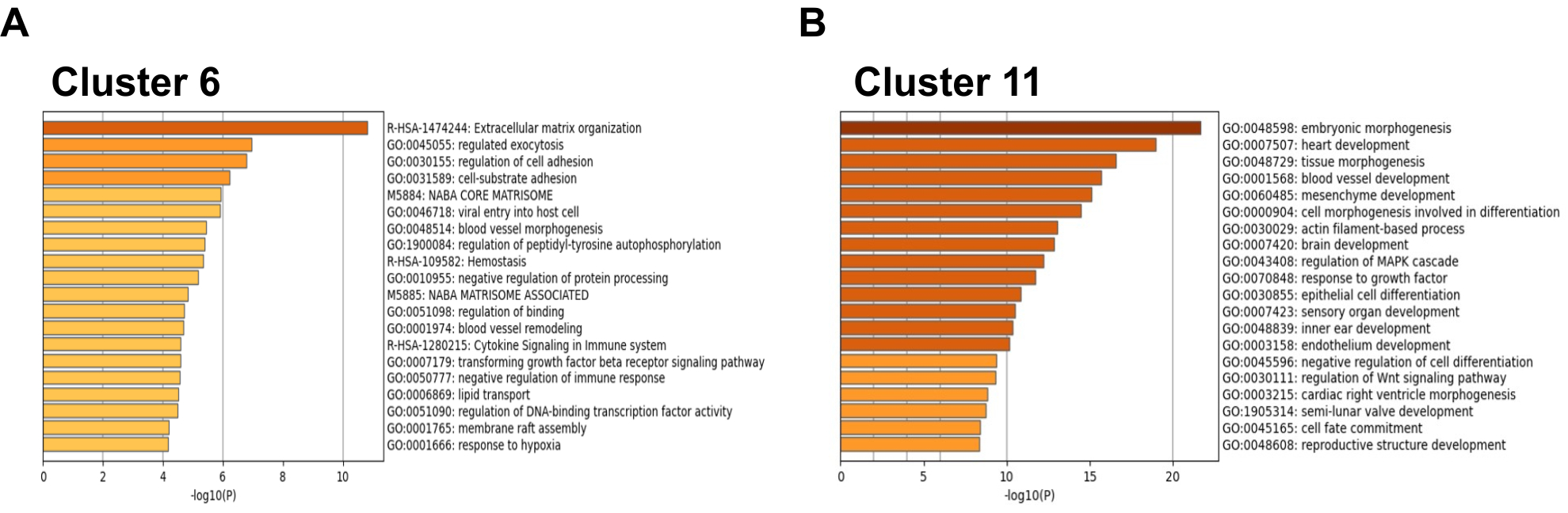

Supplement: Supplementary file 7 — Additional file 7. Cluster 6 and Cluster 11 of metascape analysis. Metascape analysis revealed a cluster with genes downregulated (cluster 6) in hiPSC-ECs as compared to other EC types and a cluster of genes upregulated (cluster 11) as compared to other EC types. The figure summarizes the gene ontologies of the genes found in the respective clusters. [file 13287_2022_2924_MOESM7_ESM.jpg]

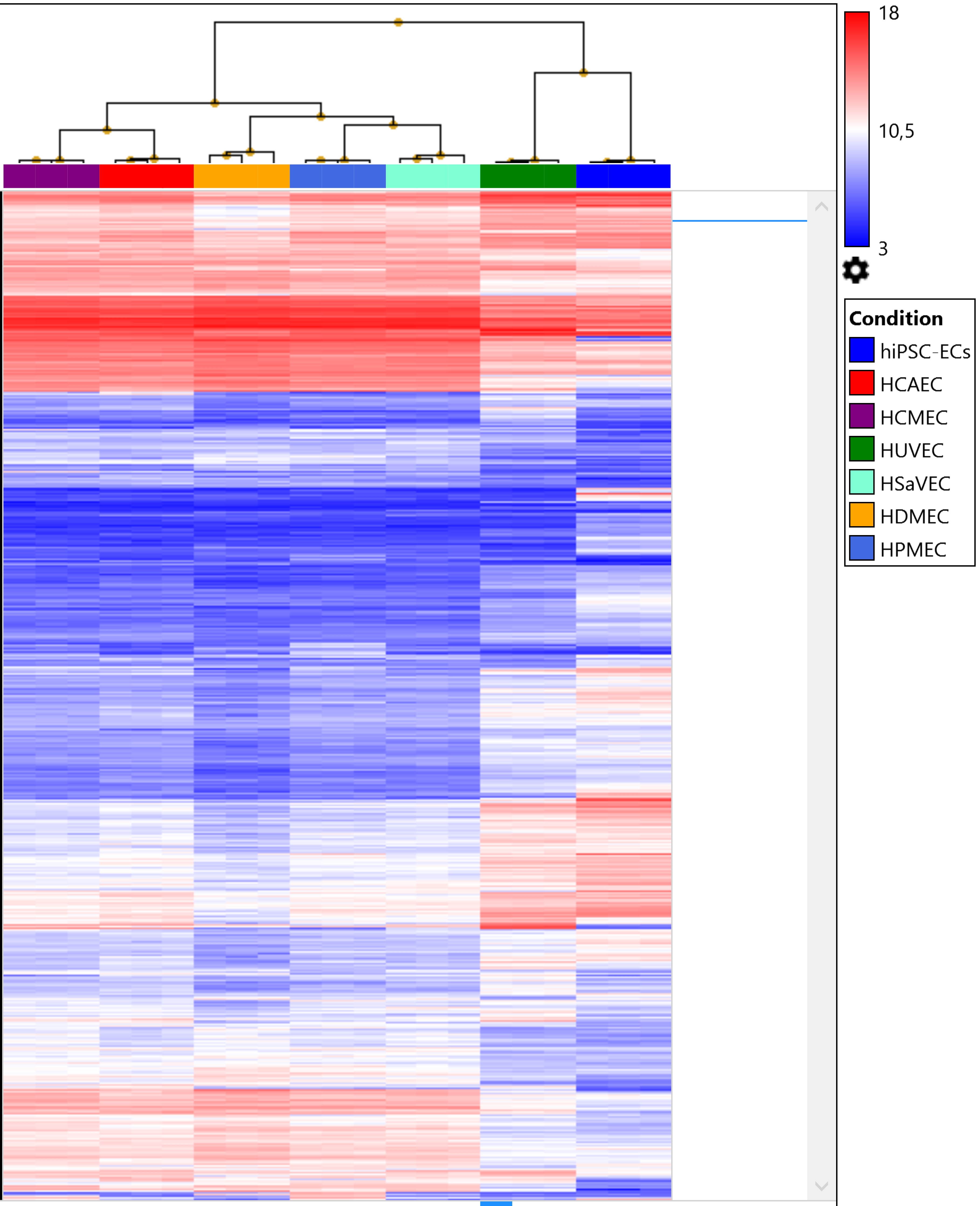

Supplement: Supplementary file 8 — Additional file 8. Heatmap of transcriptomic profiling of different types of endothelial cells. [file 13287_2022_2924_MOESM8_ESM.jpg]

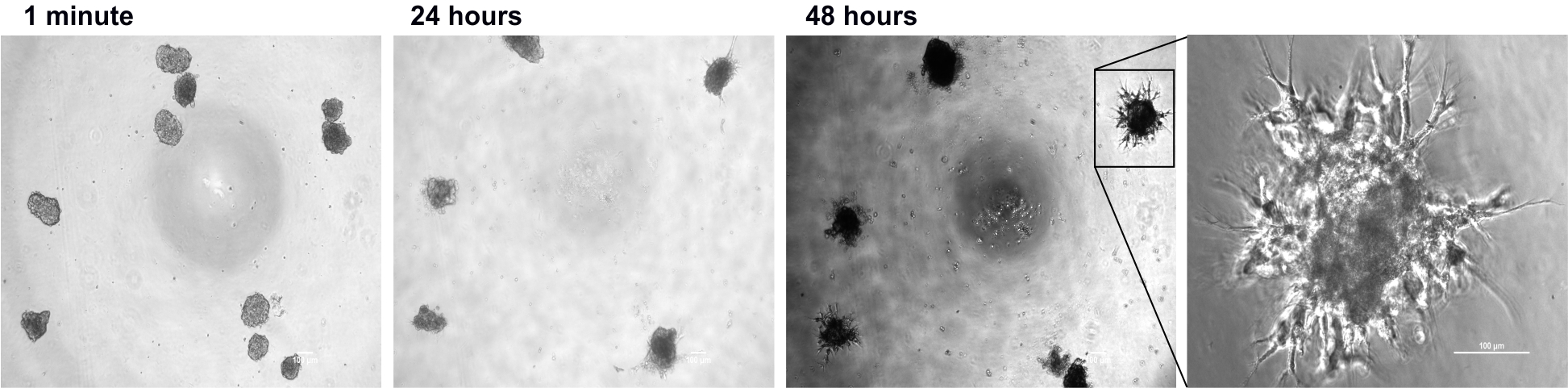

Supplement: Supplementary file 9 — Additional file 9. Morphology of hiPSC-EC clusters in 3D culture. [file 13287_2022_2924_MOESM9_ESM.jpg]

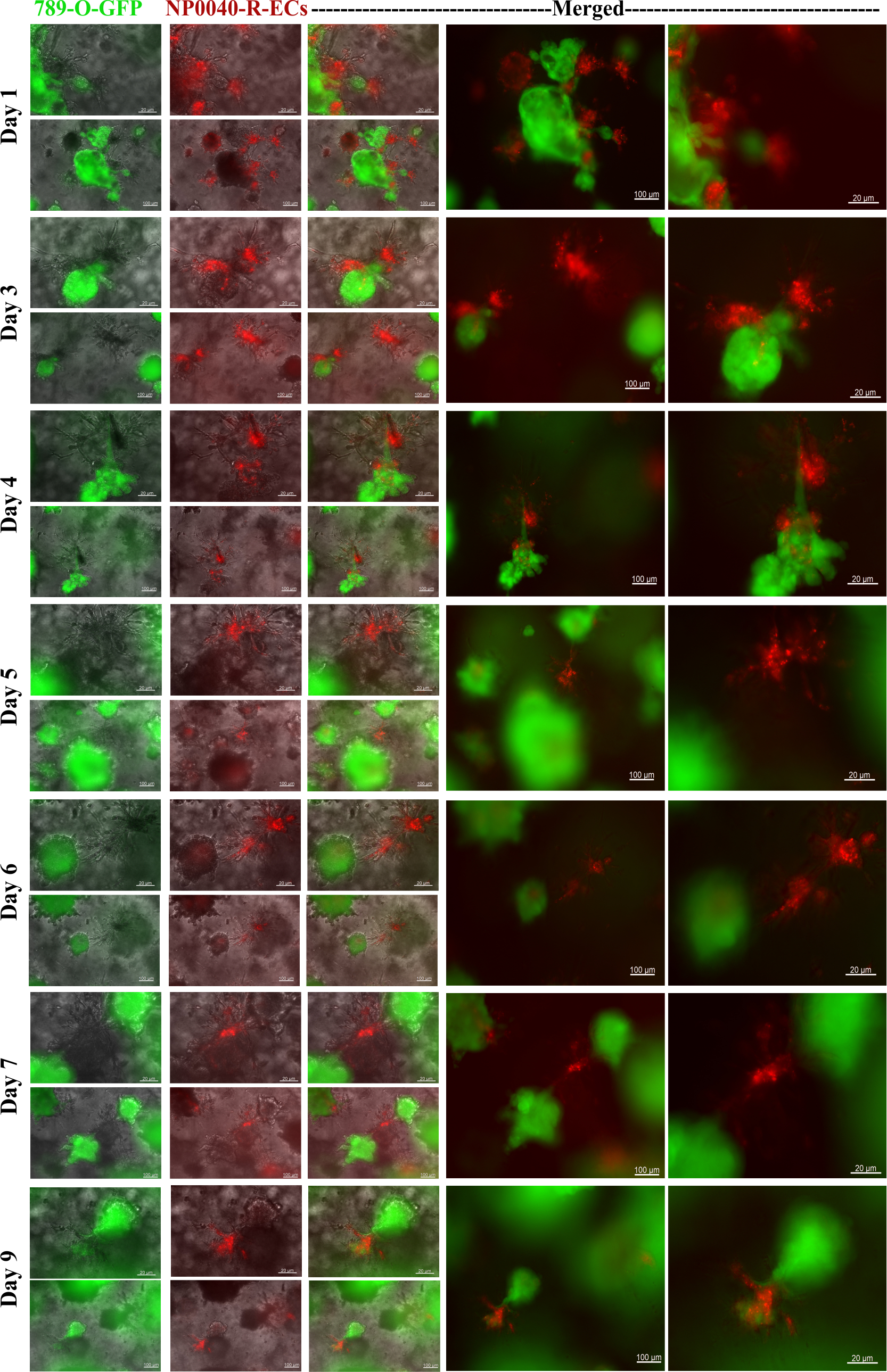

Supplement: Supplementary file 10 — Additional file 10. Morphology of red fluorescent hiPSC-EC clusters cultivated in a 3D cell culture model together with green fluorescent renal carcinoma cells. [file 13287_2022_2924_MOESM10_ESM.jpg]

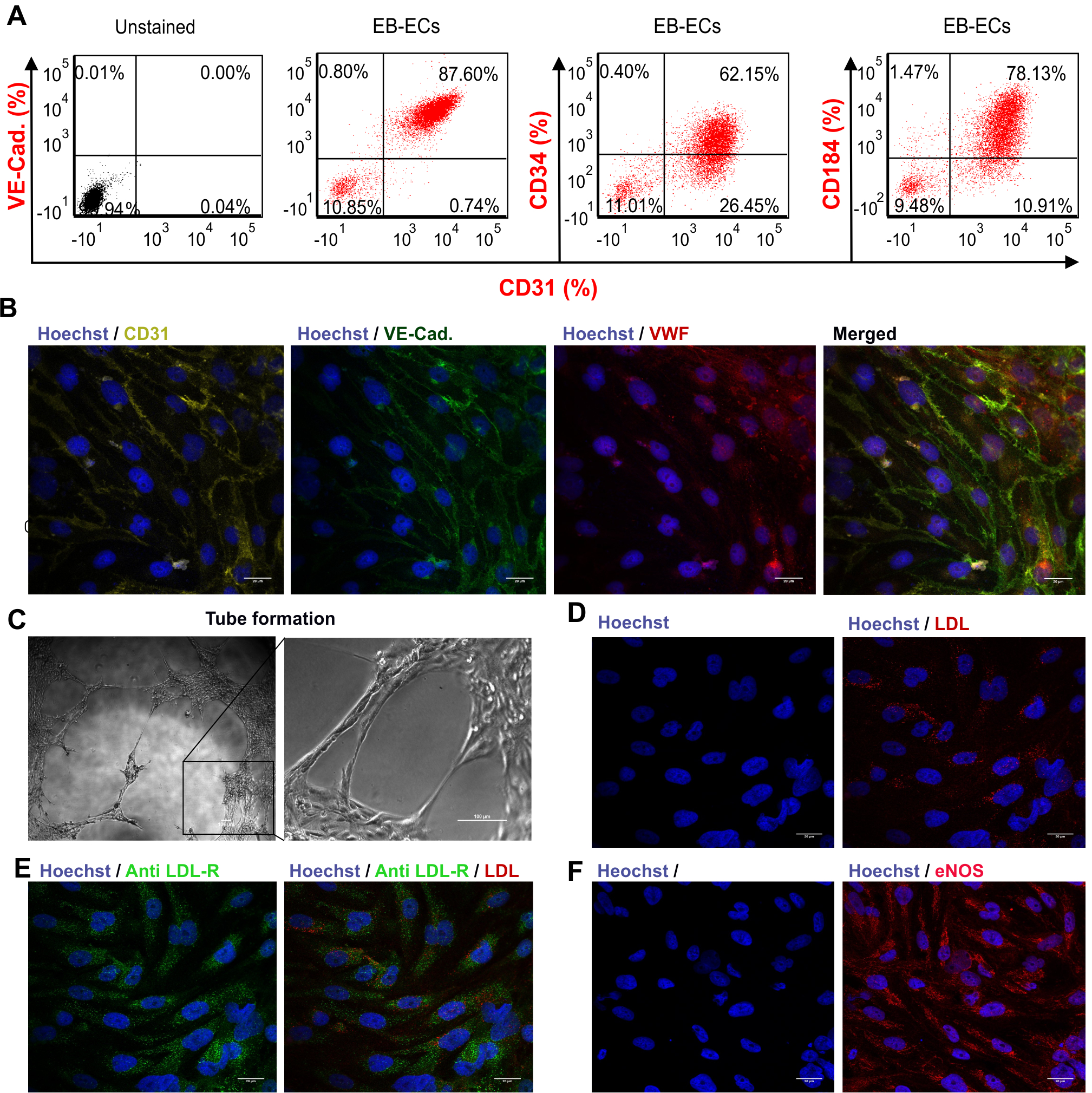

Supplement: Supplementary file 11 — Additional file 11:Analysis of hiPSC-ECs generated in a 3D bioreactor. [file 13287_2022_2924_MOESM11_ESM.jpg]
